# Increased frequencies of CD8^+^CD57^+^ T cells are associated with antibody neutralization breadth against HIV in viraemic controllers

**DOI:** 10.7448/IAS.19.1.21136

**Published:** 2016-12-09

**Authors:** Christine D Palmer, Marisol Romero-Tejeda, Eileen P Scully, Ainsley Lockhart, Michael S Seaman, Ariel Goldenthal, Alicja Piechocka-Trocha, Bruce D Walker, Lori B Chibnik, Stephanie Jost, Filippos Porichis

**Affiliations:** 1 Ragon Institute of MGH, MIT and Harvard, Cambridge, MA, USA; 2 Division of Infectious Diseases, Department of Internal Medicine, Brigham and Women’s Hospital, Boston, MA, USA; 3 Beth Israel Deaconess Medical Center Boston, MA, USA; 4 Harvard Medical School, Boston, MA, USA; 5 Howard Hughes Medical Institute, Chevy Chase, MD, USA; 6 Program in Translational Neuropsychiatric Genomics, Department of Neurology, Brigham and Women’s Hospital Boston, MA, USA; 7 Department of Epidemiology, Harvard T.H. Chan School of Public Health, Boston, MA, USA

**Keywords:** HIV, broadly neutralizing antibody, T cells, immune monitoring, biomarker, immune signature, viral load

## Abstract

**Introduction:**

An effective prophylactic vaccine against HIV will need to elicit antibody responses capable of recognizing and neutralizing rapidly evolving antigenic regions. The immunologic milieu associated with development of neutralizing antibody breadth remains to be fully defined. In this study, we sought to identify immunological signatures associated with neutralization breadth in HIV controllers. We applied an immune monitoring approach to analyze markers of T cell and myeloid cell activation by flow cytometry, comparing broad neutralizers with low- and non-neutralizers using multivariate and univariate analyses.

**Methods:**

Antibody neutralization breadth was determined, and cryopreserved peripheral blood mononuclear cells were stained for T cell and myeloid cell activation markers. Subjects were grouped according to neutralization breadth, and T cell and myeloid cell activation was analyzed by partial least squares discriminant analysis to determine immune signatures associated with high neutralization breadth.

**Results:**

We show that neutralization breadth in HIV viraemic controllers (VC) was strongly associated with increased frequencies of CD8^+^CD57^+^ T cells and that this association was independent of viral load, CD4 count and time since HIV diagnosis.

**Conclusions:**

Our data show elevated frequencies of CD8^+^CD57^+^ T cells in VC who develop neutralization breadth against HIV. This immune signature could serve as a potential biomarker of neutralization breadth and should be further investigated in other HIV-positive cohorts and in HIV vaccine trials.

## Introduction

Protective immunity elicited by currently licensed vaccines relies on the generation of neutralizing antibodies against conserved antigenic regions of the specific pathogens targeted by the vaccine [1]. In the case of HIV, an effective vaccine would need to induce antibody responses capable of recognizing and neutralizing rapidly evolving antigenic regions [2], and thus far, such antibodies have not been elicited in sufficient levels in human HIV vaccine trials [3–5]. Although HIV infection leads to the generation of HIV-specific antibodies, in particular against components of the HIV envelope (Env), these antibodies are largely non-neutralizing, appear to have little effect on viral load (VL), and any strain-specific neutralizing effects that do develop are likely to contribute to viral evolution and escape (reviewed in [1,6]). This continuous arms race between the immune system and HIV can, in some individuals, lead to the development of antibodies that are able to neutralize a broad range of different viral strains [6–9]. Isolation and characterization of such broadly neutralizing antibodies (bNAbs) revealed that these antibodies are highly somatically mutated [10] and carry insertions, deletions or long complementary determining regions [10–12] that make it difficult to elicit such antibodies via conventional immunization strategies [13]. While development of such bNAbs in the setting of chronic infection does not necessarily confer clinical benefit to the individual in whom they are induced, a vaccine that elicits this type of breadth should have substantial protective efficacy for uninfected persons.

Since the initial identification and isolation of HIV Env-reactive neutralizing antibodies [10,14,15], a large number of potent bNAbs have been cloned [6–9,16]. In fact, several recent studies have shown therapeutic efficacy of infused bNAbs in humanized mice [17,18], non-human primates [19,20] and humans [21]. While such therapeutic approaches hold great promise for efforts towards a cure and have prompted proposals for new therapeutic approaches using vectored immunoprophylaxis (VIP) [22], a substantial effort has been directed towards designing effective vaccination approaches to elicit bNAbs able to protect against HIV infection.

Vaccination strategies targeted towards eliciting bNAbs include delivery of such antibodies via VIP [13,23], sequential immunization to mimic the antigenic evolution needed to drive generation of bNAbs [1,6] and “mosaic” immunogen design based on the structure of bNAbs and their ligands [24]. The anticipated success of such vaccination approaches in inducing HIV-specific bNAbs is supported by the fact that there is no evidence for genetic predisposition to produce bNAbs [25] and that production of bNAbs seems to be linked to the initial Env sequence encountered by the immune system during early infection [26–28]. Successful evaluation of neutralization breadth of vaccine-elicited antibodies *in vitro* will require standardized assessment of these antibodies against a global panel of HIV Env reference strains [29]. Identification of surrogate immunologic markers associated with development of neutralization breadth would facilitate screening of candidate immunogens and may also provide insights into the immunologic milieu required for development of these responses.

In this study, we examined a cohort of HIV viraemic controllers (VC) in whom routine immunologic screening had been performed and neutralization breadth against a standard reference panel of 11 clade B Tier 2/3 Env pseudoviruses had been determined, with the goal of identifying immune signatures associated with the detection of neutralization breadth. We analyzed data on T cell and myeloid cell activation by standardized flow cytometry panels and compared broad neutralizers with low- and non-neutralizers using multivariate and univariate analyses. We demonstrate that neutralization breadth in VC was strongly associated with increased frequencies of CD8^+^CD57^+^ T cells independent of VL, CD4 count or duration of infection. This immune signature suggests an association between CD8 T cell function and development of neutralization breadth and identifies a potential biomarker for immune responses associated with increased neutralization breadth.

## Methods

### Ethics, subject characteristics and clinical diagnostics

This research is in compliance with the Helsinki Declaration. Subjects gave written, informed consent prior to enrolment through institutional review board-approved protocols at Massachusetts General Hospital (MGH). HIV-positive patients with undetectable plasma viral load and ≤2000 copies/ml in the absence of combination antiretroviral therapy (cART) were identified as elite controllers (EC) and viraemic controllers (VC), respectively [30]. HIV testing was performed by the Department of Microbiology at MGH using an Abbott Architect and a fourth-generation HIV Ab/Ag combo kit (Abbott Laboratories, Abbott Park, IL, USA). HIV quantitative VLs were performed on a COBAS^®^ AmpliPrep Instrument and COBAS^®^ TaqMan^®^ 48 Analyzer (Roche Molecular Diagnostics, Pleasanton, CA, USA). CD4 counts were assessed at the Clinical Flow Cytometry Laboratory at MGH using a Multitest™ kit and BD FACSCanto™ flow cytometer (BD Biosciences, San Jose, CA, USA). Subject demographics including frequencies of protective HLA-B alleles are shown in Table 1.

**Table 1:** Subject demographics

Subject characteristics	Neutralizers	Low-neutralizers	Non-neutralizers	Statistics	*p*-value	Total
Subjects (*n*)	14	18	33			65
Age range min-max (years)	26–64	30–67	31–67			26–67
Average age (years±SD)	47±11	52±10	50±9	One-way ANOVA	*p=*0.43	50±9
Male (*n*, %)	12, 86%	16, 89%	27, 82%	Chi-square	*p=*0.37	55, 85%
Race/ethnicity (*n*, %)						
African American	7, 50%	5, 28%	7, 21%			19, 29%
Asian	0, 0%	0, 0%	0, 0%			0, 0%
Caucasians	6, 43%	13, 72%	24, 73%	Chi-square	*p=*0.13	43, 66%
Hispanic	0, 0%	0, 0%	2, 6%			2, 3%
Other/unknown	1, 7%	0, 0%	0, 0%			1, 2%
Neutralization breadth (average, range)	7.9, 5–11	2.0, 1–4	0.0,0	B	B	2, 0–11
ART naïve (*n*, %)	7, 50%	12, 67%	18, 55%	Chi-square	*p=*0.28	37, 57%
Time since diagnosis (years)	18	19	16	One-way ANOVA	*p=*0.93	17.4
CD4 count (cells/µl)	737	659	796	Kruskal–Wallis	*p=*0.5	761
Protective HLA-B genotype (*n*, %)						
All subjects (*n*=65) B*27 ∣ B*57	4, 29%	7, 39%	19, 58%	Chi-square	*p=*0.11	30, 46%
VC only (*n*=41) B*27 ∣ B*57	4, 31%	4, 33%	8, 50%	Chi-square	*p=*0.5	16, 39%
Median viral load (copies/ml)						
All subjects, EC & VC (*n*=65)	400	87	67	Kruskal–Wallis	***p<0.010***	141
VC only (n=41)	400	304	205	Kruskal–Wallis	*p=*0.5	321
Viral control category (*n*, %)						
Elite	1, 7%	6, 33%	17, 52%	Chi-square	***p<0.050***	24, 37%
Viremic	13, 93%	12, 67%	16, 48%			41, 63%

p values in bold indicate significant differences.

### Reagents and samples

Peripheral blood samples were drawn into acid citrate dextrose vacutainer tubes or standard therapeutic phlebotomy whole blood collection bags for large blood donations. Peripheral blood mononuclear cells (PBMCs) were isolated by density gradient centrifugation as previously described [31] and cryopreserved in 10% dimethyl sulfoxide and 90% heat-inactivated foetal bovine serum (FBS). Samples were processed, and plasma and PBMCs cryopreserved within six hours of phlebotomy to ensure high sample quality and to avoid alteration in cellular functions that might impair the integrity of our results [32]. Cryopreserved samples were thawed and washed twice with RPMI 1640 Medium supplemented with 10% FBS (both Sigma-Aldrich) prior to analysis by flow cytometry.

### Flow cytometry

PBMCs were stained with antibody panels testing for T cell activation [33] and monocyte/DC characteristics [34] as previously described. Details of antibodies and stains used in each panel are listed in Supplementary File 1. Cells were fixed with Fix/Perm Medium (Invitrogen) and washed prior to acquisition on an LSRII flow cytometer using FACSDiva™ software (BD). Cytometer settings were kept consistent by tracking laser voltages using UltraRainbow Fluorescent Particles (Spherotech, Inc., Lake Forest, IL, USA). Compensation settings were assessed using CompBead™ particles (BD) and compensation calculated and applied in FACSDiva™ software. Samples were analyzed using FlowJo (Tree Star, Inc., Ashland, OR, USA).

### Determination of neutralizing antibody breadth

Patient plasma samples were heat-inactivated (56°C for 1 hour) and tested for neutralizing activity using a luciferase-based assay in TZM.bl cells as previously described [35]. Briefly, three-fold dilutions of plasma samples starting at a 1:20 dilution were performed in duplicate. Env-pseudotyped viruses were added to the plasma dilutions at a pre-determined titre to produce measurable infection and incubated for one hour at 37°C. TZM.bl cells were then added at 1×10^4^/well and plates incubated for 48 hours. Cells were lysed for two minutes with Bright-Glo luciferase reagent (Promega, Madison, WI, USA), and supernatant measured for luciferase activity using a Victor 3 luminometer (Perkin Elmer, Waltham, MA, USA). The 50% inhibitory dose (ID_50_) was calculated as the plasma dilution that resulted in a 50% reduction in relative luminescence units compared with virus control wells. All plasma samples were assayed against a standard reference panel of 11 clade B Tier 2/3 Env pseudoviruses [36]. Plasma neutralizing activity against each HIV Env pseudovirus was scored as positive, when ID_50_ titres were at least three-fold above Murine Leukaemia Virus negative control as previously described [37], and is summarized in Supplementary file 2.

### Stratification of patients according to their neutralization breadth

HIV controllers (EC: *n*=21; VC: *n*=41; total *n*=65) were classified according to anti-HIV antibody breadth. Subjects with 0 breadth were classified as non-neutralizers (*n*=33). Neutralization breadth in the remaining 32 subjects ranged from 1 to 11 (out of 11 clade B Tier 2/3 viruses), with a median of 4. Accordingly, this group was further subdivided according to the median, and subjects with breadth of 1 to 4 were classified as low-neutralizers (*n*=18), and subjects with breadth ≥5 were classified as high neutralizers (neutralizers; *n*=14). Similar stratifications have been applied previously [37–41]. Neutralization groups were matched for gender, age, ethnicity, time since HIV diagnosis and prior use of cART (Table 1).

### Statistical analyses

Statistical analyses were performed using GraphPad Prism 6 (GraphPad Software, Inc., La Jolla, CA, USA) and JMP^®^ 11.2.0 (SAS Institute Inc., Cary, NC, USA). Gaussian sample distributions were assessed by Shapiro–Wilk normality test. Contingency distributions were analyzed by Chi-square test. Two-way comparisons were performed using two-tailed *t*-test or Mann–Whitney test. Group comparisons were performed by One-way ANOVA with Holm-Sidak's multiple comparison correction or Kruskal–Wallis test with Dunn's multiple comparisons correction. Spearman and Pearson rank analyses were performed to assess correlations. Results were considered significant at *p<*0.05 and indicated as follows: *p<0.05, **p<0.01, ***p<0.001. Partial least square discriminant analysis (PLSDA) [42] with stepwise variable selection was used to determine multivariate immunological profiles to distinguish neutralization groups. Partial least square (PLS) regression transforms a set of correlated explanatory variables into a new set of uncorrelated “latent” (i.e. combined) variables and determines a multi-linear regression model by projecting the predicted variables and their corresponding observed values into the resulting new space. Accordingly, each sample is assigned a score that can be visualized using score plots. Latent variable loading plots can then be used to identify immunological profiles that associate with the different cohorts, as described previously [43]. Prior to multivariate analysis, all data were mean centred and variance scaled. These normalization approaches are designed to reduce bias towards variables with naturally higher raw values or variance. Within-cohort cross-validation was performed by iteratively excluding random subsets during model calibration and then using those data-excluded samples to test model discrimination and *F*-ratio rankings.

## Results

### Study design

**Figure 1:**
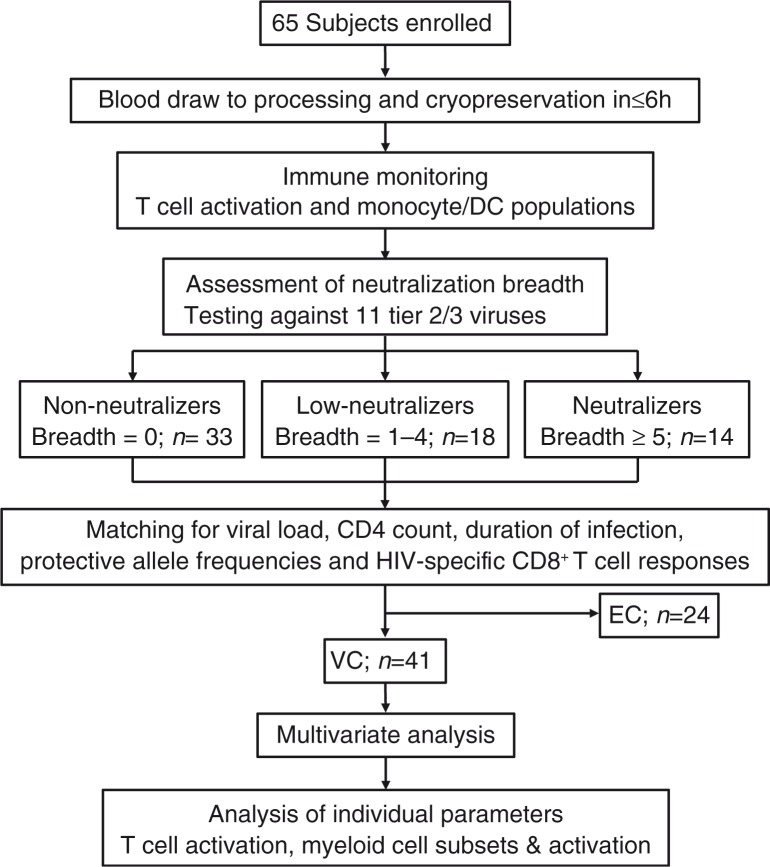
Flow-chart depicting study design.

We used the study design presented in Figure 1 to investigate 65 HIV controllers. Determination of neutralizing antibody breadth and immune monitoring for markers of T cell and myeloid cell activation was performed on all samples. For the initial analysis, all subjects were stratified into neutralizers (breadth ≥5), low-neutralizers (breadth 1–4) and non-neutralizers (breadth 0). While median VL was significantly lower in non-neutralizers compared with neutralizers (*n*=65, Table 1), overall CD4 counts, “years diagnosed,” and production of IFN-γ by PBMCs in response to clade-B HIV peptides by Enzyme-Linked ImmunoSpot (ELISPOT) did not differ between neutralization groups (data not shown). Higher proportions of subjects generating bNAbs in VC versus EC are in accordance with previous reports [44], and the observed differences in VL and HLA-B*27 and B*57 allele frequencies between neutralizers and non-neutralizers in this cohort reflected the differential distribution of EC and VC between neutralization groups (Table 1).

In line with previous studies [45], increased T cell activation as assessed by frequencies of CD8^+^CD38^+^, CD8^+^CD38^+^HLA-DR^+^, CD4^+^CD38^+^ and CD4^+^CD38^+^HLA-DR^+^ T cells correlated positively with median VL in this cohort (Supplementary file 3A, *n*=65). In contrast, these markers of T cell activation did not correlate with neutralization breadth (data not shown). Neutralization groups within VC (*n*=41) did not differ in median VL, CD4 count and “years diagnosed” (Supplementary file 3B, *n*=41). In order to avoid VL effects on immune activation skewing analyses of immune signatures associated with neutralization breadth, EC were excluded (Figure 1) and subsequent analyses were performed comparing neutralization groups in VC only, using EC as a comparator group where appropriate.

### Multivariate analysis of T cell and myeloid immune markers identifies immune signatures associated with neutralization breadth

HIV infection is associated with increased markers of T cell activation, particularly in viraemic patients [45]. Since immune responses *in vivo* are dependent on many different cellular interactions, we used PLSDA [42] to determine multivariate immunological profiles that best distinguished neutralization groups. Model predictions to classify subjects according to neutralization breadth were performed with stepwise addition of variables to ascertain the minimum number of variables needed to achieve high specificity. Variables were added based on *F*-ratio, which shows the model mean square divided by the error mean square and indicates whether the model differs significantly from a model where all predicted values are the response mean (Table 2).

**Table 2:** PLSDA stepwise variable selection

Variable	*F*-ratio	Prob>*F*
CD8^+^CD57^+^	19.3	0.00008
CD14^dim^CD16^+^	6.4	0.01576
CD8^+^CD25^+^	4.0	0.05246
CD14^dim^CD16^+^ CX3CR1 MFI	0.9	0.34417
mDC CD80 MFI	0.9	0.33338
CD8^+^HLA-DR^+^	0.4	0.52539
CD8^+^CD38^+^	0.3	0.56660
mDC CD80^+^	0.2	0.66598
CD14^+^CD16^+^	0.1	0.79906
CD4^+^CD57^+^	0.1	0.81195
CD4^+^CD38^+^	0.0	0.96123
mDC CD86 MFI	0.0	0.97346

**Figure 2:**
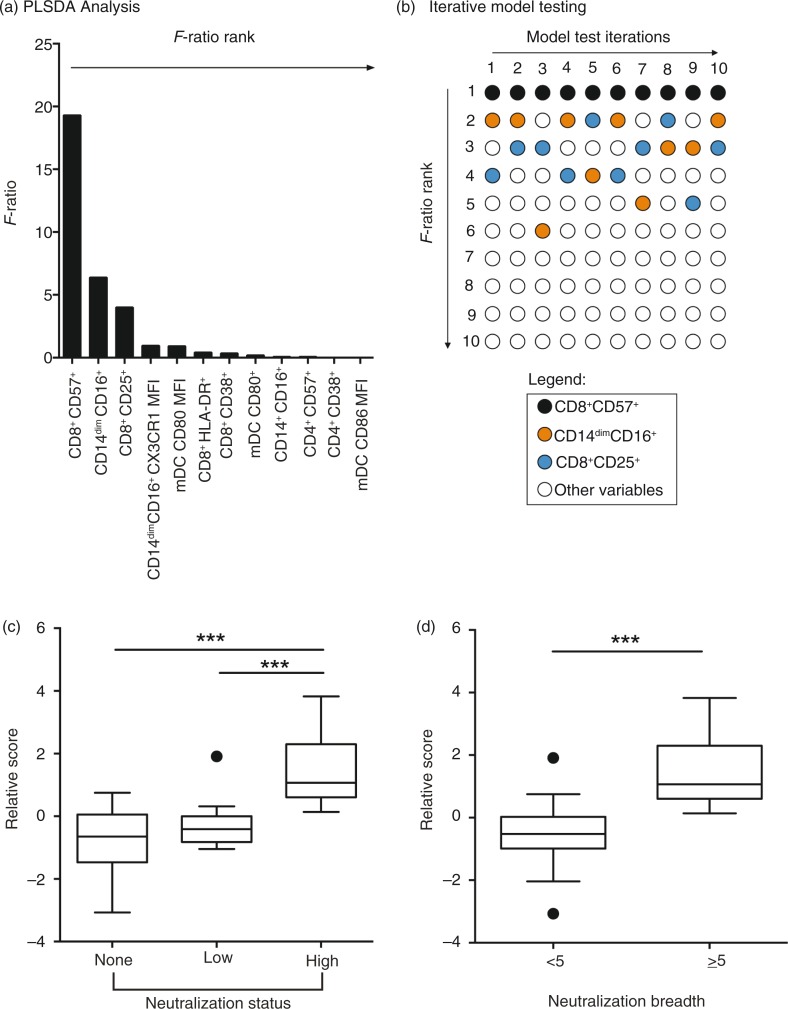
Stepwise variable selection by PLSDA allows separation of neutralization groups with combined T cell and myeloid cell data. (a) *F*-ratios for each individual variable are shown (*n*=41). (b) Iterative model testing shows ranking of immune variables by *F*-ratio for 10 iterations with 10 different random subsets of the cohort (*n*=31 per iteration). Model test iterations are shown from left to right (top), and variable ranking based on *F*-ratio from 1to 10 are shown from top to bottom. (c) PLSDA was performed including three top variables, and separation of subjects are shown in Tukey box and whiskers plots with group medians for VC subjects (*n*=41). (d) PLSDA was performed including three top variables. Graph shows separation of subjects by combined variable score on the y-axis by neutralization breadth <5 versus ≥breadth.

Consideration of *F*-ratios and *p*-values suggested that frequency of CD8^+^CD57^+^ T cells was the most prominent immune signature associated with neutralization breadth in these VC (Table 2, Figure 2a), with frequencies of CD14^dim^CD16^+^ monocytes and CD8^+^CD25^+^ T cells ranking second and third, respectively.

In order to verify that the observed immune signature was not driven by a small subset of subjects in this cohort, we performed iterative tests in which 10 different subjects were randomly removed for each of 10 iterations, and ranking of variables by *F*-ratio were assessed (Figure 2b). Iterative testing confirmed that frequency of CD8^+^CD57^+^ T cells was ranked first for every iteration of the model (Figure 2b), confirming this variable identified by PLSDA for the entire cohort. PLSDA performed incorporating the top three ranking variables (Table 2) resulted in clear separation of neutralizers (breadth ≥ 5) from low-neutralizers (breadth=1–4) and non-neutralizers (breadth=0; Figure 2c, *n*=41). Based on these observations, we stratified patients into two groups by neutralization breadth of <5 or ≥5. Accordingly, PLSDA using three variables also showed clear separation by neutralization breadth <5 versus ≥5 (Figure 2d). Inclusion of variables 1 to 3 with the highest *F*-ratios and significant *p*-values (Table 2) resulted in correct classification of subjects by neutralization group (< or ≥5 breadth) with 87.8% specificity. It is important to note at this point that due to unavailability of a second, separate controller cohort, specificity in this model was calculated from the cohort in which the model was developed, leading to potential over-fit and an inflated estimate. Nonetheless, these data demonstrate that analytical approaches combining three variables allow separation of subjects with neutralization breadth of <5 and ≥5 in this cohort.

### Increased frequency of CD8^+^CD57^+^ T cells in high neutralizers compared to low- and non-neutralizers

**Figure 3:**
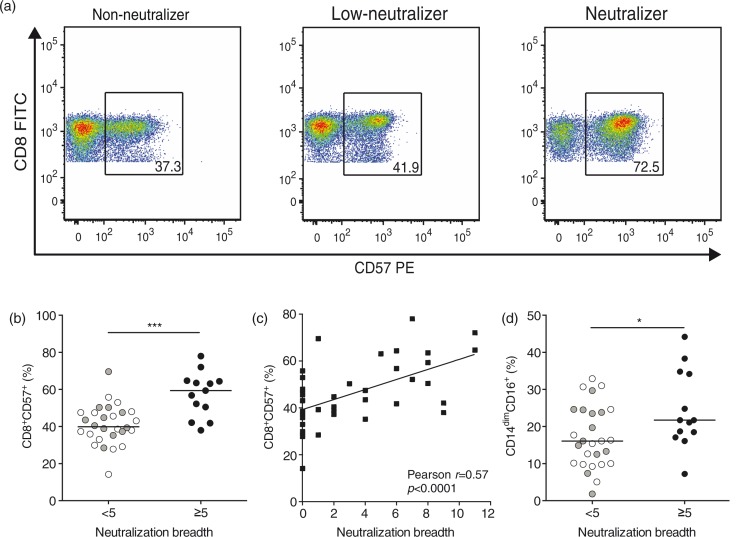
Increased frequencies of CD8^+^CD57^+^ T cells in neutralizers. (a) Representative dot plots showing frequencies of CD8^+^CD57^+^ T cells in non-neutralizers, low-neutralizers and neutralizers. (b) Frequency of CD8^+^CD57^+^ T cells in VC (*n*=41) with group median is shown for breadth <5 (non-neutralizers in white circles, low-neutralizers in grey circles, *n*=28) versus breadth ≥5 (neutralizers, black circles, *n*=13). (c) Pearson correlation of neutralization breadth with frequency of CD8^+^CD57^+^ T cells (VC only, *n*=41). (d) Frequency of CD14^dim^CD16^+^ monocytes in VC (*n*=41) with group median is shown for breadth <5 (non-neutralizers in white circles, low-neutralizers in grey circles, *n*=28) versus breadth ≥5 (neutralizers, black circles, *n*=13).

In order to validate variables identified by PLSDA, we performed univariate analyses of markers of T cell activation (CD38, HLA-DR, CD25, CD69), senescence/terminal differentiation (CD57) and proliferation (Ki67) on CD4^+^ and CD8^+^ T cells in VC (*n*=41). Frequencies of CD8^+^CD57^+^ T cells were significantly higher in neutralizers compared to either low-neutralizers or non-neutralizers in VC (Figure 3a, b; *n*=41) and correlated positively with neutralization breadth (Figure 3c, *r*=0.57, *p<*0.001; *n*=41). Frequencies of CD8^+^CD57^+^ T cells did not correlate with “years diagnosed” (*r*=0.08, *p=*0.6, *n*=41) and were thus not a measure of duration of infection. Furthermore, investigations into correlations of CD8^+^CD57^+^ T cell frequencies with viral control revealed that there was no correlation with VL (Supplementary file 4A; *r*=0.23, *p=*0.15; *n*=41) and that CD8^+^CD57^+^ T cell frequency did not differ between EC and VC (Supplementary file 4B; *p=*0.33; VC *n*=41, EC *n*=24) or between subjects with or without protective HLA-B genotypes (Supplementary file 4C; *p=*0.5 for *n*=65; *p=*0.8 for *n*=41). Frequencies of CD8^+^CD57^+^ T cells did not correlate with VL even when EC, who have considerably lower VL compared with VC, were included (*n*=65, data not shown). Confirmed clade B infection status (by sequencing or ELISPOT response to Clade B peptides) was unavailable for two VC subjects. However, removal of these subjects from the above analyses (*n*=39) did not alter any of the observed differences or levels of significance. In line with variables identified by PLSDA, no other T cell activation or proliferation markers investigated in this study revealed any significant differences between neutralization groups in VC (data not shown). However, Spearman rank analyses revealed a positive correlation between frequency of CD8^+^CD57^+^ T cells with total CD8^+^ T cell frequency (*r*=0.33, *p<*0.01, *n*=41), and negative correlations with total CD4^+^ T cell (*r*=−0.27, *p<*0.05, *n*=41) and CD4^+^CD25^+^ T cell frequencies (*r*=−0.27, *p<*0.05, *n*=41).

Univariate analyses of myeloid markers revealed increased frequencies of CD14^dim^CD16^+^ monocytes in neutralizers (breadth ≥5) compared with low- and non-neutralizers (breadth <5; Figure 3d). Pearson correlation showed that CD14^dim^CD16^+^ monocyte frequencies correlated with frequencies of CD8^+^CD57^+^ T cells (*r*=0.34, *p<*0.05, *n*=41), but not with neutralization breadth (*r*=0.23, *p=*0.15, *n*=41). This suggests that ranking of this variable in PLSDA was due to its correlation with CD8^+^CD57^+^ T cell frequency, explaining its changing position between second and sixth rank in the model test iterations we performed (Figure 2b). Frequencies of CD8^+^CD25^+^ T cells did not differ between neutralization groups (data not shown). Taken together, univariate analyses suggest that frequency of CD8^+^CD57^+^ T cells in VC is strongly associated with neutralization breadth ≥5 independent of VL and duration of infection, and that CD8^+^CD57^+^ T cell frequencies are the main immune marker associated with neutralization breadth in this cohort of VC.

## Discussion

In this study, we investigated the relationship of immune signatures identified through routinely used immune monitoring panels with production of neutralizing antibody breadth in a cohort of HIV VC. Using multivariate and univariate analyses, we show that CD8^+^CD57^+^ T cell frequencies correlated positively with neutralization breadth. Previous studies have focused on CD4^+^ T cell characteristics associated with bNAb in HIV infection. Specifically, development of neutralization breadth was associated with increased frequencies of PD-1^+^CD4^+^ T cells in early infection [16], and with higher frequencies of class-switched antibodies in co-cultures of CXCR5^+^CD4^+^ T and B cells [39]. Others found no correlation between frequencies of peripheral T_FH_ cells with neutralization activity in untreated HIV-positive individuals [38]. Using a more generalized immune monitoring approach, our analyses identified a strong signature of increased CD8^+^CD57^+^ T cell frequencies with neutralization breadth in this cohort of HIV controllers.

CD57 is considered a marker of terminal differentiation, and expansion of CD8^+^CD57^+^ T cells was associated with decreased VLs in a study of non-controllers [46]. In light of these findings, it is interesting to note that there was no difference in CD8^+^CD57^+^ T cell frequencies between VC and EC, nor any correlation of this marker with VL in our cohort. The aforementioned study [46] used a smaller cohort than the current one and investigated T cell frequencies in the context of higher VLs (compare VL range of 2000 to 3,219,000 copies/ml, *n*=9 in [46] vs. 50 to 2355 copies/ml, *n*=41 in this study). Nonetheless, frequencies of CD8^+^CD57^+^ T cells did not differ between chronic-treated (median VL 20 copies/ml, range undetectable-581 copies/ml) and chronic-untreated subjects (median VL 4865 copies/ml, range 649 to 1.58×10^7^ copies/ml; unpublished observations), suggesting that our observation that frequencies of CD8^+^CD57^+^ T cells do not correlate with VL also hold true in individuals with high VLs in the absence of treatment.

Furthermore, our findings are in agreement with a recent study showing that frequencies of CD8^+^CD57^+^ T cells did not correlate with VL in treated and untreated patients [47]. It is nonetheless remarkable that despite the correlation of VL with neutralization breadth here (with inclusion of EC) and in other studies [8], the association of increased CD8^+^CD57^+^ T cell frequencies with neutralization breadth is independent of VL. While elevated percentages of CD8^+^CD57^+^ T cells have been described as a symptom of chronic immune activation [48] and of accelerated immune senescence in chronic HIV infection [49], several studies have also shown that CD8^+^CD57^+^ T cells elicit potent cytotoxic effector functions in HIV and other viral infections [50,51] through high expression of perforin and secretion of IFN-γ and TNF-α [52]. A recent study comparing HIV-specific T cell responses in neutralizers and non-neutralizers showed a greater breadth and magnitude of CD4^+^ T cell responses to HIV Gag by ELISPOT in neutralizers versus non-neutralizers [37], suggesting a more effective CD4 T cell response. In addition, our data showing that CD8^+^CD57^+^ T cell frequencies correlated positively with total CD8^+^ T cell frequency and negatively with CD4^+^CD25^+^ T cell frequencies may indicate that elevated frequencies of CD8^+^CD57^+^ T cells in VC with high neutralization breadth reflect reduced T regulatory and increased cytotoxic T cell frequencies in these subjects. Generation of bNAbs in treated subjects has been linked to longer periods of detectable viraemia [53], and even though overall viraemia did not correlate with CD8^+^CD57^+^ T cell frequencies or neutralization breadth in VC, it is also possible that higher frequencies of CD8^+^CD57^+^ T cells are indicative of an immune response to increased viral *diversity* driving the production of bNAbs without correlating with overall VL.

Previous studies have aimed at identifying immune signatures in early HIV infection that might predict subsequent production of bNAbs [16,39]. In contrast, this study was designed to determine immune activation signatures concurrent with neutralization breadth. Data presented by Mikell *et al*. comparing HIV-positive subjects early in infection showed no increase in CD8^+^CD57^+^ T cell frequencies in subjects that later developed bNAbs [16]. The authors argued that small sample size precluded detection of any immune signals at a statistically significant level. Using a larger cohort of chronically infected individuals with spontaneous virologic control but detectable viraemia, our study shows clear differences in CD8^+^CD57^+^ T cell frequencies in high neutralizers compared with low- and non-neutralizers, adding to this prior work. It is important to note that good sample integrity was critical for this finding, as others have shown that delay between blood collection and sample processing alters cellular responses and surface expression levels [31,32]. This was confirmed in the present cohort, as differences in CD8^+^CD57^+^ T cell frequencies were not apparent in samples where blood was left at room temperature for 18 to 24 hours prior to processing and cryopreservation. Flow cytometry has evolved from a relatively inaccessible investigative tool to a widely-used and accessible method of interrogating cellular phenotypes in laboratories across the globe [54], making it ideal as a screening tool for use in HIV vaccine trials. In addition, while studies aiming at cross-cohort comparisons are susceptible to differences in site-specific protocols and machine set-up [55,56], screening for relative differences in CD8^+^CD57^+^ T cell frequencies in a specific cohort would not require such coordinated efforts. It is important to note that our study was limited by the fact that we did not have an independent validation cohort to test our model predictions on, and that our within-cohort iterative testing is likely to be over fit and give an inflated estimate of the predictive value of this immune signature. It is, therefore, vital for this immune signature to be investigated and corroborated in additional cohorts comparing HIV-positive subjects stratified by neutralization breadth. If validated, the immune signature identified in this study could allow quick identification of subjects with neutralization breadth-associated immune signatures prior to being sent for standardized analyses for neutralization breadth and potency as previously proposed [29]. Thus far, HIV vaccine trials have not elicited sufficiently protective antibody responses, and in particular, the production of bNAbs has not been achieved by the more conventional vaccination approaches tested [3–5]. In the context of such studies, it is unlikely that the immune responses elicited by a conventional vaccine approach are comparable with those driving bNAb production in individuals that are able to control viraemia. However, in the light of two very recent exciting studies using single and sequential immunization strategies with germline-targeting immunogens to induce maturation and recombination of the VRC01 germline in mice [57,58], it is feasible that a vaccination strategy developed to mimic the natural processes leading to the evolution of bNAbs would also induce corresponding immune signatures to those observed *in situ*. In the context of the latter scenario, an immune signature associated with neutralization breadth could also be applied to pre-screen samples in vaccine trials, opening the door for cheaper and more efficient analysis of such samples prior to in-depth analysis of antibody profiles.

## Supplementary Material

Increased frequencies of CD8^+^CD57^+^ T cells are associated with antibody neutralization breadth against HIV in viraemic controllers

Increased frequencies of CD8^+^CD57^+^ T cells are associated with antibody neutralization breadth against HIV in viraemic controllers

Increased frequencies of CD8^+^CD57^+^ T cells are associated with antibody neutralization breadth against HIV in viraemic controllers

Increased frequencies of CD8^+^CD57^+^ T cells are associated with antibody neutralization breadth against HIV in viraemic controllers
