# Supplementary material for: Increased frequencies of CD8+CD57+ T cells are associated with antibody neutralization breadth against HIV in viraemic controllers
Source: J Int AIDS Soc. 2016 Dec 9;19(1):21136. doi: 10.7448/IAS.19.1.21136 (PMC5149708; doi:10.7448/IAS.19.1.21136)
Supplement: Increased frequencies of CD8+CD57+ T cells are associated with antibody neutralization breadth against HIV in viraemic controllers [file JIAS-19-21136-s001.pdf]

Additional File 1:

Panel 1: T cell activation

Antibody/Stain	Species	Colour	Compary
LIVE/DEAD® Fixable Blue Dead Cell Stain	--	Blue	Invitrogen
anti-CD3	mouse	BV711	BioLegend
anti-CD4	mouse	Qdot605	Invitrogen
anti-CD8	mouse	FITC	BD
anti-CD38	mouse	PE-Cy7	BD
anti-HLD-DR	mouse	APC-H7	BD
anti-CD57	mouse	PE	BD
anti-CD25	mouse	BV421	BD
anti-CD69	mouse	PercP	BD
anti-Ki67	mouse	AlexaFluor647	BD

Panel 2: Monocyte/DC characteristics

Antibody/Stain	Species	Colour	Compary	
Fixable live dead stain	--	eFluor780	eBioscience	
anti-CD3	mouse	FITC	BD	Dump channel
anti-CD19	mouse	FITC	BD	
anti-CD56	mouse	FITC	BD	
anti-CD14	mouse	BUV395	BD	
anti-CD11c	mouse	PE cf594	BD	
anti-CD123	mouse	PE-Cy7	BD	
anti-CD16	mouse	BV510	BioLegend	
anti-CCR2	mouse	PE	BioLegend	
anti-CX3CR1	rat	APC	BioLegend	
anti-CD80	mouse	BV421	BioLegend	
anti-CD86	mouse	BV605	BioLegend	
anti-HLA-DR	mouse	BV785	BioLegend	
