# Supplementary material for: Increased frequencies of CD8+CD57+ T cells are associated with antibody neutralization breadth against HIV in viraemic controllers
Source: J Int AIDS Soc. 2016 Dec 9;19(1):21136. doi: 10.7448/IAS.19.1.21136 (PMC5149708; doi:10.7448/IAS.19.1.21136)
Supplement: Increased frequencies of CD8+CD57+ T cells are associated with antibody neutralization breadth against HIV in viraemic controllers [file JIAS-19-21136-s002.pdf]

Additional File 2:

Virus	Tier	Positive responses* n (%)		
		Neutralizers	Low-neutralizers	Non-neutralizer
QH0692.42	2	6 (42.9)	1 (5.6)	0 (0)
SC422661.8	2	14 (100)	6 (33.3)	0 (0)
PVO.4	2/3	9 (64.3)	3 (17.6)	0 (0)
TRO.11	2	13 (92.9)	7 (38.9)	0 (0)
AC10.0.29	2	11 (78.6)	4 (22.2)	0 (0)
RHPA4259.7	2	11 (78.6)	4 (22.2)	0 (0)
THRO4156.18	2	7 (50)	1 (5.6)	0 (0)
REJO4541.67	2	12 (85.7)	10 (55.6)	0 (0)
TRJO4551.58	2/3	7 (50)	0 (0)	0 (0)
WITO4160.33	2	7 (50)	3 (16.7)	0 (0)
CAAN5342.A2	2	10 (71.4)	3 (16.7)	0 (0)

* ID_{50} titres ≥3-fold above Murine Leukaemia Virus negative control
