# Supplementary material for: Increased frequencies of CD8+CD57+ T cells are associated with antibody neutralization breadth against HIV in viraemic controllers
Source: J Int AIDS Soc. 2016 Dec 9;19(1):21136. doi: 10.7448/IAS.19.1.21136 (PMC5149708; doi:10.7448/IAS.19.1.21136)
Supplement: Increased frequencies of CD8+CD57+ T cells are associated with antibody neutralization breadth against HIV in viraemic controllers [file JIAS-19-21136-s003.pdf]

Additional File 3:

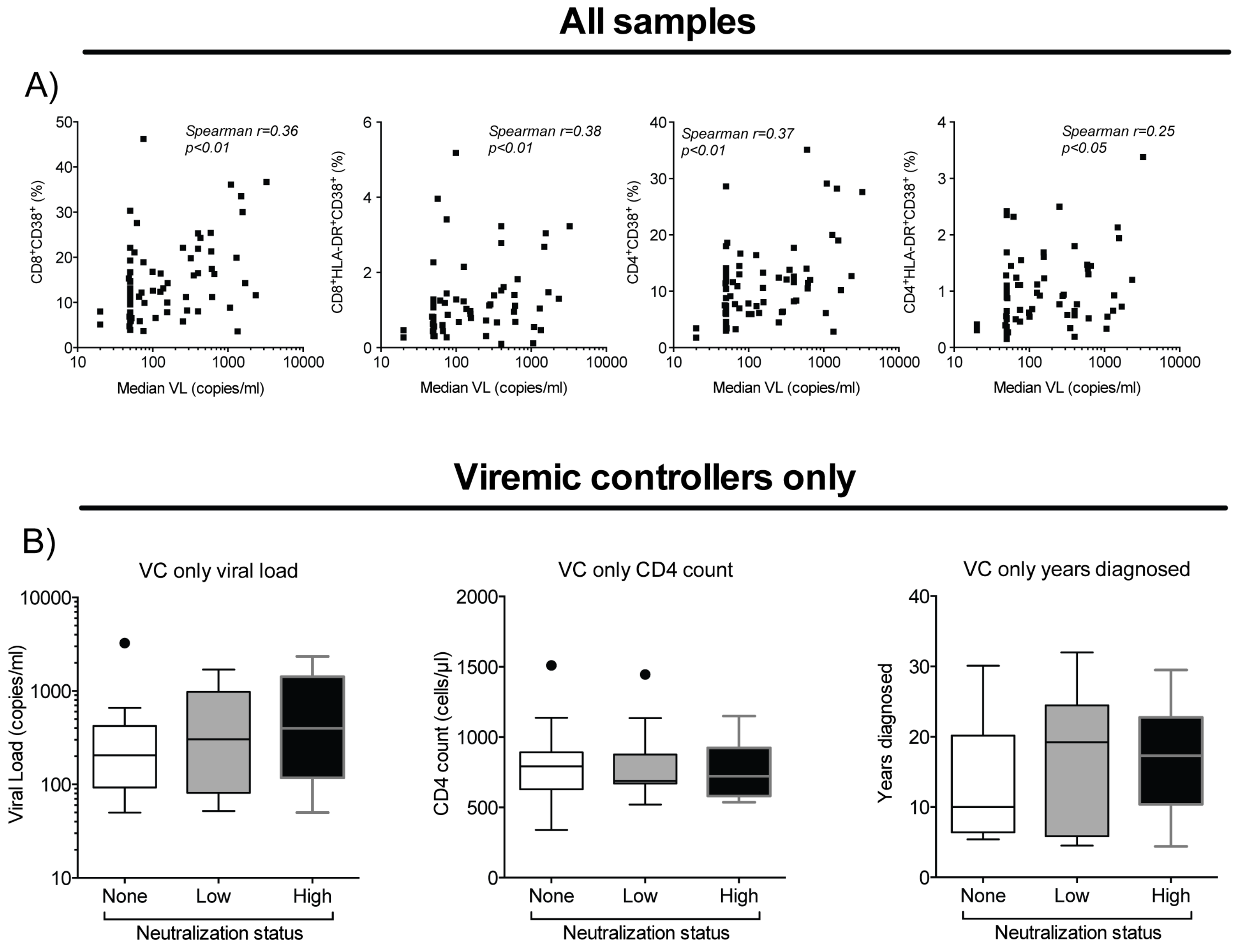

Additional File 3: Exclusion of EC reduces VL effects on T cell activation. Subjects (n=65) were grouped into neutralizers (n=14), low-neutralizers (n=18) and non-neutralizers (n=33). **A)** Correlation analyses of median viral load (VL) with $CD8^+CD38^+$, $CD8^+CD38^+HLA\text{-}DR^+$, $CD4^+CD38^+$, and $CD4^+CD38^+HLA\text{-}DR^+$ T cells were performed for all subjects (n=65). **B)** Tukey box and whiskers plots with group medians are shown for VC subjects only (n=41). Graphs show viral load (left graph), total CD4 count (middle graph), and 'years diagnosed' (right graph) in non-neutralizers (clear, n=16), low-neutralizers (grey, n=12) and neutralizers (black, n=13). Statistical analyses comparing non-neutralizers, low-neutralizers and neutralizer were performed using Kruskal-Wallis test with Dunn's multiple comparisons test. Correlations were performed using Spearman rank analyses. Results were considered significant at $p<0.05$.
