# Supplementary material for: Increased frequencies of CD8+CD57+ T cells are associated with antibody neutralization breadth against HIV in viraemic controllers
Source: J Int AIDS Soc. 2016 Dec 9;19(1):21136. doi: 10.7448/IAS.19.1.21136 (PMC5149708; doi:10.7448/IAS.19.1.21136)
Supplement: Increased frequencies of CD8+CD57+ T cells are associated with antibody neutralization breadth against HIV in viraemic controllers [file JIAS-19-21136-s004.pdf]

Additional File 4:

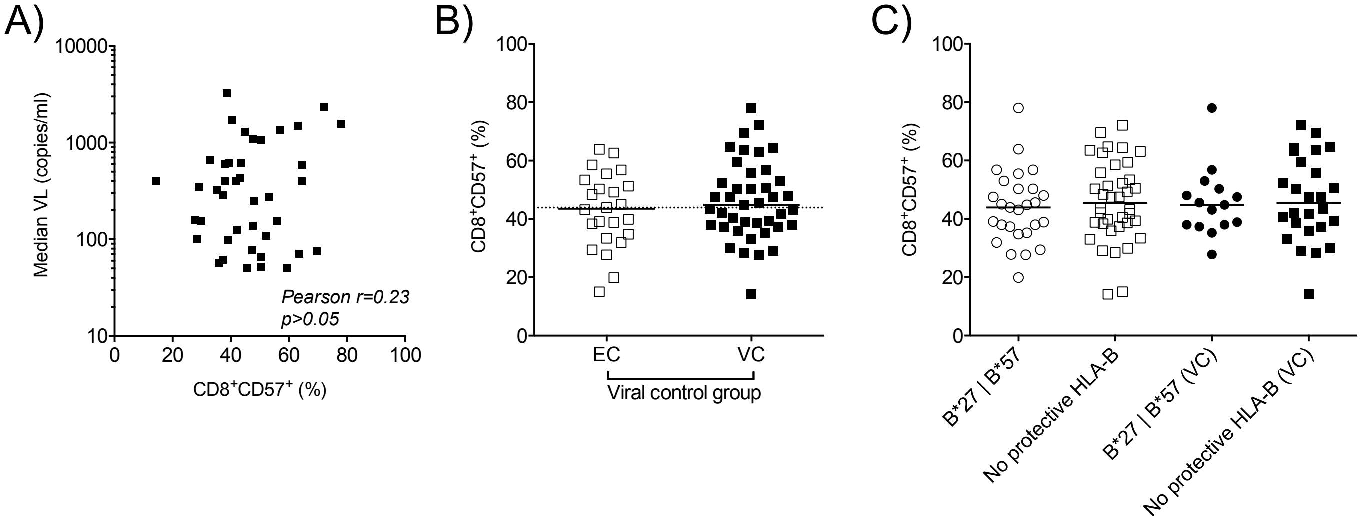

Additional File 4: Frequency of CD8+CD57+ T cells does not correlate with viral load or control status. A) Correlation analyses of median viral load (VL) with $CD8^{+}CD57^{+}$ T cells were performed for VC only (n=41). **B)** Dot plots with group medians are shown for frequency of CD8+CD57+ T cells in VC subjects (open squares, n=41) and EC subjects (black squares, n=24). Overall group median (n=65) is indicated by the dotted line (43.9%). **C)** Dot plots with group medians are shown for frequency of CD8+CD57+ T cells in subjects with any protective HLA-B genotype in the entire cohort (n=65; open circles = HLA-B*27 and/or B*57, open squares = no protective HLA-B genotype) and VC subjects (n=41; black circles = HLA-B*27 and/or B*57, black squares = no protective HLA-B genotype). Statistical analyses comparing VC and EC or HLA groupings were performed using an unpaired t-test. Correlations were performed using Spearman rank analyses. Results were considered significant at $p<0.05$.
